# Multicentered analysis of percutaneous sclerotherapies in venous malformations of the face

**DOI:** 10.3389/fmed.2022.1066412

**Published:** 2022-12-13

**Authors:** Vanessa F. Schmidt, Max Masthoff, Constantin Goldann, Richard Brill, Peter B. Sporns, Laura Segger, Victor Schulze-Zachau, Martin Takes, Michael Köhler, Sinan Deniz, Osman Öcal, Nabeel Mansour, Muzaffer Reha Ümütlü, Mwivano Dunstan Shemwetta, Balowa Musa Baraka, Eric M. Mbuguje, Azza A. Naif, Ofonime Ukweh, Max Seidensticker, Jens Ricke, Bernhard Gebauer, Walter A. Wohlgemuth, Moritz Wildgruber

**Affiliations:** ^1^Department of Radiology, University Hospital, LMU Munich, Munich, Germany; ^2^Clinic for Radiology, Münster University Hospital, Münster, Germany; ^3^Clinic and Policlinic of Radiology, Martin Luther University Halle-Wittenberg, Halle, Germany; ^4^Department of Neuroradiology, Clinic of Radiology and Nuclear Medicine, University Hospital Basel, Basel, Switzerland; ^5^Department of Diagnostic and Interventional Neuroradiology, University Medical Center Hamburg-Eppendorf, Hamburg, Germany; ^6^Department of Radiology, Charité – University Medicine Berlin, Berlin, Germany; ^7^Department of Interventional Radiology, Clinic of Radiology and Nuclear Medicine, University Hospital Basel, Basel, Switzerland; ^8^Department of Radiology, Muhimbili University of Health and Allied Sciences, Dar es Salaam, Tanzania; ^9^Department of Radiology, University of Calabar, Calabar, Nigeria

**Keywords:** VM, slow-flow, face, sclerotherapy, outcome

## Abstract

**Objectives:**

To evaluate the safety and outcome of image-guided sclerotherapy for treating venous malformations (VMs) of the face.

**Materials and methods:**

A multicenter cohort of 68 patients with VMs primarily affecting the face was retrospectively investigated. In total, 142 image-guided sclerotherapies were performed using gelified ethanol and/or polidocanol. Clinical and imaging findings were assessed to evaluate clinical response, lesion size reduction, and complication rates. Sub-analyses of complication rates depending on type and injected volume of the sclerosant as well as of pediatric versus adult patient groups were conducted.

**Results:**

Mean number of procedures per patient was 2.1 (±1.7) and mean follow-up consisted of 8.7 months (±6.8 months). Clinical response (*n* = 58) revealed a partial relief of symptoms in 70.7% (41/58), 13/58 patients (22.4%) presented symptom-free while only 4/58 patients (6.9%) reported no improvement. Post-treatment imaging (*n* = 52) revealed an overall objective response rate of 86.5% (45/52). The total complication rate was 10.6% (15/142) including 4.2% (7/142) major complications, mostly (14/15, 93.3%) resolved by conservative means. In one case, a mild facial palsy persisted over time. The complication rate in the gelified ethanol subgroup was significantly higher compared to polidocanol and to the combination of both sclerosants (23.5 vs. 6.0 vs. 8.3%, *p* = 0.01). No significant differences in complications between the pediatric and the adult subgroup were observed (12.1 vs. 9.2%, *p* = 0.57). Clinical response did not correlate with lesion size reduction on magnetic resonance imaging (MRI).

**Conclusion:**

Image-guided sclerotherapy is effective for treating VMs of the face. Clinical response is not necessarily associated with size reduction on imaging. Despite the complex anatomy of this location, the procedures are safe for both adults and children.

## Introduction

According to the classification of the International Society for the Study of Vascular Anomalies (ISSVA) (1, 2) vascular malformations are divided in slow-flow and high-flow lesions, related to the underlying flow pattern, which is crucial both for treatment decision and the prognosis (3). Slow-flow malformations represent the majority (>90%) of vascular malformations and among these, venous malformations (VMs) are the most common type (4). VMs are composed of a dilated, dysplastic, and hemodynamically non-functional venous-like network presenting as compressible, bluish, non-pulsatile, and soft with expanding skin and subcutaneous tissue (5). In many cases, VMs grow proportionally during childhood remaining unnoticed for years prior to symptomatic clinical presentation (6). While possibly occurring at any part of the body, VMs frequently affect the head and neck area including the face (7). As the complex anatomy of the face is based on multiple small functional units containing dense innervation and limited soft tissue coverage, therapy of these lesions often has a relevant risk for nerve injury, necrosis, and aesthetic disfigurement (8).

The purpose of this multicenter study was to evaluate the safety and outcome of image-guided sclerotherapy with ethanol gel and/or polidocanol for the treatment of VMs affecting the face.

## Materials and methods

### Study design

The present retrospective multicenter study was approved by the local ethics committee (University Hospital, LMU Munich, protocol No. 21-0943, 10/06/2021) and was performed in accordance with the Declaration of the World Medical Association (WMA). All patients were recruited from Interdisciplinary Vascular Anomalies Centers at six tertiary care university hospitals in Germany, Switzerland, and Tanzania. Data collection was performed using electronic patient records as well as the Picture Archiving and Communication System (PACS) searching for corresponding diagnosis related groups (DRGs). VMs were diagnosed based on the combination of patient history, physical examination, and imaging using magnetic resonance imaging (MRI) as well as duplex ultrasound (9). The angiographic classification was performed according to Puig et al. (10). All malformations predominantly affected the face, patients with lesions involving the neck or head without the face were excluded. In addition, patients who underwent diagnostic workup only without invasive treatment were excluded. The indications for image-guided sclerotherapy were pain, swelling, bleeding, recurring infections, repetitive thrombosis and/or other blood coagulopathy, aesthetic disfigurement as well as accompanying functional impairment (e.g., insufficient breathing, jaw motional restriction, epiphora, exophthalmos, and facial paresthesia).

### Procedural details

Interventional treatment was conducted under general anesthesia. Sclerotherapy was performed under real-time ultrasound and fluoroscopic guidance using gelified ethanol (Sclerogel^®^, Balt Germany GmbH & Co. KG, Düsseldorf, Germany) and/or 2–3% polidocanol foam (Aethoxysclerol^®^, Kreussler & Co. GmbH, Wiesbaden, Germany; ratio of polidocanol to sterile air 1:4). Gelified alcohol was used in cases of rapid venous drainage toward larger draining veins due to its higher viscosity. For sclerotherapy ultrasound guidance was applied to gain access to the VM. After placing a 20/21G needle within the lesion, venous blood was aspirated for verification of intralesional position and contrast injection under fluoroscopy was performed in order to detect rapid venous drainage into the deep venous system and classify the lesion. Subsequently the sclerosant was injected in majority of cases under ultrasound control, either until the lesion was properly covered or dislocation of sclerosant toward the deep venous system occurred. Only in case of lesions with fast venous drainage or drainage toward susceptible areas like the brain, the filling defect technique using fluoroscopy was additionally used. E.g., for orbital malformations, additional fluoroscopic was routinely applied. Patients were discharged at day 2–5 following the procedure, with low molecular weight heparin routinely administered for 7 days, in order to prevent potential acute postinterventional symptoms, especially those caused by local thrombophlebitis. Repetitive sclerotherapy procedures were performed depending on the extent of the lesion, response to therapy, and course of clinical symptomatology.

### Follow-up

In the six centers involved the patients were seen within a standardized follow-up regime. The first clinical follow-up was conducted at 1–3 months after each sclerotherapy session including repeated contrast-enhanced MRI examination. In case of insufficient improvement of symptoms or residually perfused lesion(s) being present, an additional sclerotherapy session was performed. In case of no additional treatment the next follow-up was scheduled at 6 months, again comprising a clinical examination as well as contrast-enhanced MRI. Hereafter, additional follow-ups were performed annually.

### Outcome evaluation

Data analysis was conducted to evaluate demographics and to define lesion classification, clinical response, objective response (imaging), and complication rates. Clinical response at follow-up was measured using the following grading scale: symptom-free, partial relief of symptoms, no improvement of symptoms, and clinical progression despite sclerotherapy. Objective response was assessed by changes in lesion size using pre-procedural MR images compared to those obtained at terminal follow-up after the last sclerotherapy. Imaging findings were classified into the following four categories: complete response (CR, 100% lesion size reduction), partial response (PR, 30% lesion size reduction), stable disease (SD, neither PR nor PD criteria met), progressive disease (PD, 20% lesion size increase) (11, 12). For the size assessment of the VMs on delayed-phase contrast-enhanced fat-saturated T1-weighted images, the largest lesion diameter in one imaging plane was used, comparable to the response evaluation criteria in solid tumors (RECIST) (11). Peri- and post-procedural complications were classified into minor and major adverse events (AE) according to the Cardiovascular and Interventional Radiological Society of Europe (CIRSE) classification (13) as well as analyzed depending on the type and the injected volume of the sclerosant and between the adult and pediatric subgroup. A comparison of clinical and objective response was performed.

### Statistical analyses

Descriptive statistics were used to analyze the distribution of patients among the different categories. Kolmogorov–Smirnov (K-S) test was used for the assessment of normality. Data are presented as means (±SD) in case of normal distribution or as medians (range, minimum–maximum) for skewed distribution. Sub-analyses were performed using the Pearson’s Chi-squared test for categorial data and the Mann–Whitney U test for metric data. Statistical testing was conducted using SPSS (version 26.0, IBM Corp., USA), with *p* < 0.05 considered as significant.

## Results

### Patients characteristics

A total of 68 consecutive patients, 30 males and 38 females, with VMs of the face underwent a total of 142 image-guided sclerotherapies between 2010 and 2021 (Table 1). The median age was 18.0 years (range, 0.7–64 years) at treatment initiation while 31/68 (45.6%) have been pediatric cases (<18 years). In general, the 68 VMs presented with frontal (4/68, 5.9%), temporal (11/68, 16.2%), orbital (5/68, 7.4%), auricular (4/68, 5.9%), nasal (4/68, 5.9%), buccal (54/68, 79.4%), mental (12/68, 17.6%), and labial (17/68, 25.0%) involvement. Part of the cohort (24/68, 35.3%) presented with extensive lesions extending to more than 1 of these anatomical areas. Thereof, one VM (1/68, 1.5%) included 6 areas and one 4 areas, while 12/68 patients (17.6%) presented lesions extending to 3 areas and 10/68 patients (14.7%) to 2 areas. None of the lesions extended into retroorbital areas and none of the patients presented with VMs associated with other anomalies (such as Klippel-Trenaunay syndrome). Puig et al.’s classification (10) showed mostly type I (28/68, 41.2%) and type II (27/68, 39.7%) while 13/68 lesions (19.1%) were categorized as type III. Both therapy-naive patients (36/68, 52.9%) and patients having undergone previous invasive treatments (32/68, 47.1%) by debulking surgery (16/68, 23.5%), sclerotherapy (16/68, 23.5%), or laser-therapy (6/68, 8.8%) without sufficient symptom improvement, were included. Clinical follow-up and MR imaging was obtained for 52/68 patients (76.5%). Regarding the remaining 16 patients, 10/68 (14.7%) have been lost to follow-up while 6/68 patients (8.8%) were children <6 years of age; in the latter cases, only a clinical follow-up was performed, as the aim was to avoid the anesthesia required for MRI and the clinical responses had been sufficient. The mean follow-up period after the last treatment session was 8.7 (±6.8) months for the total cohort.

**TABLE 1:** Patient characteristics of the study cohort.

Characteristic		Cohort (total, *n* = 68)	Cohort (at terminal follow-up, *n* = 52^1^/58^2^)
Age at diagnosis	Median (range)	10.5 (0–59)	
Men		30 (44.1%)	
Lesion size (ml)	Median (range)	18.5 (1.5–582.9)	7.0 (1.0–359.1)^1^
Max. lesion diameter (mm)	Median (range)	40.0 (5.0–82.0)	21.5 (3.0–65.0)^1^
**Puig classification**			
Type I		28 (41.2%)	
Type II		27 (39.7%)	
Type III		13 (19.1%)	
Type IV		0 (0.0%)	
**Involved anatomical areas**			
Frontal		4 (5.9%)	
Orbital		5 (7.4%)	
Nasal		4 (5.9%)	
Temporal		11 (16.2%)	
Buccal		54 (79.4%)	
Labial		17 (25.0%)	
Mental		12 (17.6%)	
Auricular		4 (5.9%)	
**Treatment rationales**			
Pain		41 (60.3%)	
Swelling		63 (92.6%)	
Thrombosis		7 (10.3%)	
Cosmetic disfigurement		41 (60.3%)	
Functional limitation		10 (14.7%)	
Impaired swallowing^3^		7 (10.3%)	
Impaired breathing^3^		3 (4.4%)	
Accompanying sequelae		5 (7.4%)	
Exophthalmus		3 (4.4%)	
Blurry visualization		1 (1.5%)	
Sialadenitis		1 (1.5%)	
**Symptom graduation**			
None		0 (0.0%)	13 (19.1%)^2^
Light		3 (4.4%)	26 (38.2%)^2^
Moderate		22 (32.4%)	19 (27.9%)^2^
Strong		36 (52.9%)	0 (0.0%)^2^
Very strong		7 (10.3%)	0 (0.0%)^2^

Max., maximum; SD, standard deviation; define *n* = 52^1^/58^2^, related to the available cohort size with MRI (1) and clinical (2) data at terminal follow-up; ^3^related to swelling of the cheek/lips, however, without objective obstruction of the upper airways.

### Procedural characteristics

The mean number of image-guided sclerotherapies per patient was 2.1 (±1.7), for details see Table 2. Thereby, 66/142 procedures (46.5%) were performed in children. A total of 84/142 sclerotherapies (59.2%) were performed using polidocanol foam with a mean injected volume of 4.9 ml (±3.7) while 34/142 treatments (23.9%) were performed using gelified ethanol with a mean injected volume of 1.5 ml (±0.9). Of the 34 latter, lipidiol was mixed to gelified ethanol (1:1) in 9 cases resulting in a mean injected volume of 0.7 ml (±0.6). With regard to the 25/142 (17.6%) treatments with pure gelified ethanol, the mean injected volume was 1.8 ml (±0.9). A sequential combination of both sclerosants, polidocanol foam, and gelified ethanol, with a mean injected volume of 4.5 ml (±4.9) and 2.2 ml (±0.8), respectively, was used in 24/142 procedures (16.9%). The median duration of hospitalization was 3.0 days (range, 2–5 days).

**TABLE 2:** Procedural data of the study cohort.

Characteristic	Cohort (total, *n* = 68)
Age at treatment initiation median (range)	18.0 (0.7–64)
**Total number of serial procedures**	
1	35 (51.5%)
2	17 (25.0%)
3	6 (8.8%)
4	4 (5.9%)
5	1 (1.5%)
6	2 (2.9%)
7	2 (2.9%)
8	1 (1.5%)

SD, standard deviation.

### Clinical response

Final clinical follow-up (after last sclerotherapy) revealed an overall response of 54/58 patients (93.1%) including mainly partial relief of symptoms (41/58, 70.7%) and symptom-free presentation (13/58, 22.4%). There was no improvement of symptoms in 4/58 patients (6.9%) at the last obtained follow-up. No patient presented with clinical progression or relapse of symptoms following sclerotherapy.

### Imaging response

**FIGURE 1:**
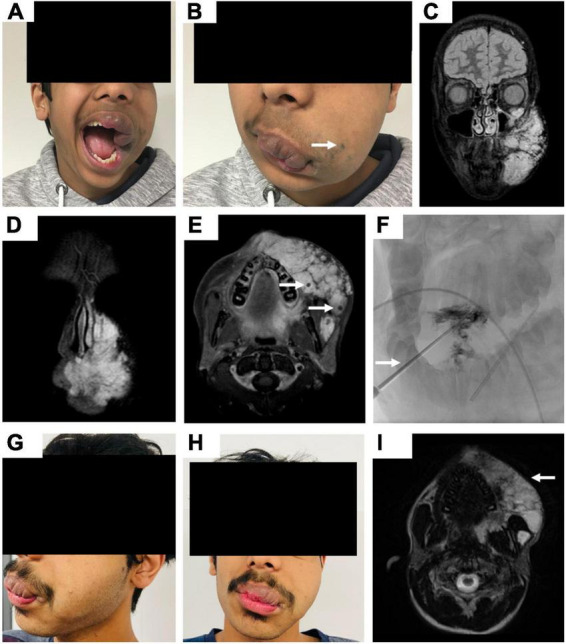
Fourteen-year-old male patient with a venous malformation (VM) primarily affecting the nasal, buccal, mental, and labial area of the left face. **(A,B)** Clinical photographs demonstrate the appearance of the patient with extensive swelling in the areas affected by the VM and enormous labial deformation on the left side. Superficial bluish discoloration of the skin is marked by an arrow. **(C–E)** Coronar and axial fat-saturated T2-weighted MR images present typical hyperintense signal of the facial VM with multiple hypointense spots representing phleboliths (arrows). **(F)** Periprocedural digital subtraction angiography (DSA) images while successful percutaneous sclerotherapy of oropharyngeal compartments, the arrow marks the oral inserted angiocatheter. **(G,H)** Clinical photographs show the appearance of the patient 3 years post sclerotherapy with reduced swelling of the affected areas. The patients’ lip closure can be performed completely as well as no functional problems with food intake occur. **(I)** In parallel, post-treatment axial T2-weighted MR image reveals a reduced volume of the VM (arrow).

After the last sclerotherapy session, the lesion size evaluation of the post-treatment MRI at terminal follow-up compared to pre-treatment imaging revealed CR in 6/52 patients (11.5%), PR in 39/52 patients (75.0%), and SD in 7/52 patients (13.5%). This resulted in an overall objective response rate of 86.5% (45/52), see Figure 1.

### Safety and complications

Peri- and postprocedural complications were reported after 15/142 sclerotherapies (10.6%, CIRSE grade 1–4). During one procedure (1/130, 0.7%, CIRSE grade 1), persistent venous bleeding occurred which was resolved after application of suprarenin and a sponge sealant patch coated with human fibrinogen/thrombin as well as manual compression. The postprocedural complications included local skin necroses/ulcerations (2/142, 1.4%, CIRSE grade 3) at the treated area of malformation, in one patient with temporary bleeding, both conservatively resolved by intensified wound management. Further adverse events involved prolonged swelling at injection side (4/142, 2.8% CIRSE grade 2) entailing elongated postprocedural observation, abscess formations successfully treated with antibiotics during prolonged hospitalization (2/142, 1.4%, CIRSE grade 3) as well as mild superficial inflammations (4/142, 2.8%, CIRSE grade 2), the latter not resulting in additional measures. One patient suffered from thrombophlebitis beyond the treated area of malformation (1/142, 0.7%, CIRSE grade 3) 1 week after sclerotherapy. In one case a mild facial palsy occurred postprocedural (1/142, 0.7%, CIRSE grade 4) basically manifested as mouth asymmetry which partially improved but did not recover entirely. Except for the latter case, all complications were self-limited and entirely resolved with conservative means (14/15, 93.3%). Overall, the major complication rate (CIRSE grade >2) was 4.2% (6/142). In our patient cohort, no systemic procedure-related complications have been reported such as allergic reactions due to administered drugs, postinterventional syncope, nausea/vomiting, or fever. No late complications (>30 days) were reported.

### Sub-analysis of sclerosant type and volume

The sub-analysis revealed significant differences between the procedures performed with gelified ethanol compared to polidocanol foam concerning the incidence of post-procedural complications (Chi-squared test, *p* = 0.01): regarding the polidocanol subgroup (*n* = 84) a total complication rate of 6.0% (5/84) was found while in the gelified ethanol subgroup (*n* = 34) complications occurred in 23.5% (8/34). In patients with both sclerosant type used in combination (*n* = 24) a complication rate of 8.3% (2/24) was calculated. No significant differences between these subgroups according to the sclerosant used and clinical as well as imaging response were evaluated (Chi-squared test, *p* > 0.05). No significant differences between the sclerosant volume injected and the complication rates were found (Mann–Whitney U test, *p* = 0.11).

### Correlation of clinical and imaging response

The evaluation (of all patients with clinical follow-up and MRI, *n* = 52) revealed no statistically significant differences between the lesion size reduction on MRI compared to the grade of clinical response (Chi-squared test, *p* = 0.15), even though the percentage of malformations achieving CR on MRI was higher in the symptom-free group (2/11, 18.2%) than in the group with partial relief of symptoms (4/37, 10.8%), see Table 3.

**TABLE 3:** Lesion size reduction by clinical response.

	Clinical response (*n* = 52)		
	
	Symptom-free (*n* = 11)	Partial relief of symptoms (*n* = 37)	No improvement of symptoms (*n* = 4)
**Lesion size reduction (*n* = 52)**			
Complete response (*n* = 6)	2 (3.8%)	4 (7.7%)	0 (0.0%)
Partial response (*n* = 39)	9 (17.3%)	28 (53.8%)	2 (3.8%)
Stable disease (*n* = 7)	0 (0.0%)	5 (9.6%)	2 (3.8%)

SD, standard deviation; define complete response, 100% lesion size reduction; partial response, 30% lesion size reduction; stable disease, neither partial response nor progressive disease criteria met.

### Comparison of children and adults

Comparison of the sclerotherapies performed in children and adults revealed no significant differences in clinical response (Chi-squared test, *p* = 0.40) or imaging response (Chi-squared test, *p* = 0.93), respectively, for details see Table 4. In addition, there were no statistically significant differences between the complication rates (Chi-squared test, *p* = 0.57) in both groups: regarding the pediatric subgroup (*n* = 66) a total complication rate of 12.1% (8/66) was found while complications occurred in 7/76 adult patients (9.2%).

**TABLE 4:** Comparison of outcomes between pediatric and adult group.

	Pediatric group (n = 19^1^/25^2^)	Adult group (n = 33)	*P*-value
Lesion size reduction (*n* = 52)			*p* = 0.93
Complete response (*n* = 6)	2 (3.8%)^1^	4 (7.7%)	
Partial response (*n* = 39)	14 (26.9%)^1^	25 (48.1%)	
Stable disease (*n* = 7)	3 (5.8%)^1^	4 (7.7%)	
Clinical response (*n* = 58)			*p* = 0.40
Symptom-free (*n* = 13)	5 (8.6%)^2^	8 (13.8%)	
Partial relief (*n* = 41)	17 (29.3%)^2^	24 (41.4%)	
No improvement (*n* = 4)	3 (5.2%)^2^	1 (1.7%)	

SD, standard deviation; define complete response, 100% lesion size reduction; partial response, 30% lesion size reduction; stable disease, neither partial response nor progressive disease criteria met; define *n* = 19^1^/25^2^, related to the available pediatric cohort with MRI (1) and clinical (2) data at terminal follow-up.

## Discussion

In this study of VMs primarily affecting the face, a high overall clinical and objective response rate for image-guided sclerotherapy is being reported, accompanied by a low rate of major complications.

In general, several studies reported on the outcome of image-guided therapies of VMs of the head and neck (14, 15) but were rarely restricted to the face (16); consequently, the comparison of our data with the literature is limited. With respect to most of the studies in this field (17), we evaluated a large cohort while the mean number of sclerotherapies per patient of 2.1 is similar to that reported in the literature.

We were able to demonstrate that percutaneous sclerotherapy of VMs of the face using gelified ethanol and/or polidocanol foam is effective regarding clinical and imaging response which results were also comparable in the adult and pediatric subgroups, respectively. Our findings of at least a partial relief of symptoms in 93% of the patients are similar to those reported after sclerotherapy of head and neck VMs in general (17). Likewise, equivalent results were reported in the few cohorts that focused specifically on facial lesions as exemplarily shown by Spence et al. treating 32 facial VMs using bleomycin and reporting subjective improvement in 91% (16). In this cohort, in which the risk of pneumonitis and pulmonary fibrosis as well as the maximum dose limitation with repeated use of bleomycin had to be considered, a mean dose of 10.5 U was used per session in an average of 3.5 sessions, consisting of a moderate total dose of bleomycin. In the present study a similar clinical outcome could be achieved with comparable complications regarding the minor complication rate of 13% in the cohort of Spence et al. (16). Further, there are currently novel approaches such as combining directly injected bleomycin with reversible electroporation (electrosclerotherapy) to increase the effectiveness of the sclerosants thereby reducing the dose and the risk for relevant adverse events (18). In our study, postprocedural MRI revealed an overall response rate of 87%, supported by the results of previously published studies of head and neck VMs (19, 20). Regarding the correlation of symptom improvement with objective response on MRI, different results have been published. Several studies reported a positive correlation between clinical and objective findings, such as Alexander et al. in 37 venous and lymphatic head and neck malformations (21). Other data suggested that clinical improvement is not always associated with size reduction on MRI (16). Regarding our results, the percentage of malformations achieving complete a response on MRI was higher (18%) in the symptom-free group compared to the group with partial relief of symptoms (11%), though this was not confirmed by statistical significance. Thus, in our opinion, a fraction of patients presents with substantial clinical benefit after sclerotherapy despite a lack of size reduction on MRI, and MRI at follow-up should never be considered as the primary or sole measure of treatment success. Nevertheless, it should be taken into account that due to the relatively short follow-up in our cohort, MRI may not (yet) have been able to show the possibly ongoing remodeling of connective tissue in the treated lesion. In addition, when considering the outcome of this study, it should also be noted that this cohort tended to present fewer complex lesions (Puig I and II = 81% vs. Puig III and IV = 19%) even if similar distributions have been described in the literature as well (10, 22). In parallel to the correlating of clinical outcome with post-treatment MRI, there have also been valuable approaches to obtain predictive data for upcoming treatment planning from pre-treatment imaging. Goyal et al. developed their own MR classification in their series and found that the number of sclerotherapy sessions, the amounts of ethanol for each lesion, and the number of access sites increased with increasing lesion grade (23).

The present study showed a low overall complication rate of 11%, which was also similar in the adult and pediatric subgroups considered separately, with most sequalae having resolved by conservative means. This is rather low compared to published data of sclerotherapy of extracranial VMs such as summarized in the meta-analysis by De Maria et al. involving 37 head and neck studies with a local temporary complication rate of 27–57% (17). Consequently, the presented approach confirms the acceptable risk profile of both sclerosants used and makes them particularly attractive for repeated sclerotherapy sessions even in challenging anatomical locations. Nevertheless, comparing these sclerosants in relation to the incidence of complications, we found significantly more complications in the group being treated with gelified ethanol compared to polidocanol. The latter is a frequently descripted sclerosant causing lysis of vessel endothelium while showing low complication rates (24). Gelified ethanol, a composition of ethanol, is supplemented with water-insoluble cellulose derivative and embedded by a cotton wool-like network. Local adverse events, such as necrosis, temporary nerve palsy, and ethylcellulose fistulas, are reported in 12–48% of patients (25, 26), therefore, this is generally similar to our findings (24%). The hospitalization period of a median of 3 days may be considered long in some countries where sclerotherapies may even be carried out as day-care procedures, which however may also reflect differences in reimbursement systems across countries.

Further, it should be noted that the classification of complications referring to CIRSE used here for facial VMs may not specifically reflect essential relevant consequences related to this specific localization, particularly nerve damage or aesthetic disfigurement, which significantly affect patients in the long term. In our study, a singular case of mild facial palsy occurred which did not resolve completely. The mean injected volume of sclerosant in our study was rather low, which may additionally account for the overall low complication rate. More aggressive approaches can be more effective but may potentially be accompanied with more adverse events, which in our eyes should particularly be considered in anatomically challenging areas such as the face. Though there is some evidence that higher sclerosant volumes may increase the peri- and postprocedural complication rate (27), we could not confirm this relationship in our cohort. This may be due to the fact that by using several access needles, it is possible to distribute the sclerosant over larger lesion volumes avoiding local peak concentrations at the injection sites. Even if we did not analyze the exact number, the use of several puncture sites might help in the reduction of complications.

This multicenter analysis has several limitations: first, it represents a retrospective design including a lack of standardized follow-up data available for the reported patient cohort. Second, standardized disease-related questionnaires to evaluate the specific symptomatology and functional impairments were not routinely used as systematic tools for clinical response. Exemplarily, visual analog scales (VAS) to classify pain as a symptom would have been desirable for more standardized pain assessment. In general, standardized evaluation of quality of life (QoL) may be an appropriate measurement here that should be investigated in further studies using prospective study designs. There was recently published a prospective study protocol presenting QoL as primary study objective after treatment of arteriovenous malformations (28). Additionally, further approaches are being developed to standardize treatment outcome measures, such as the international core outcome set developed by Horbach et al. (29). Third, the objective response was measured by postprocedural changes in VM size as assessed by MRI. For this purpose, no standardized protocol or guidelines/recommendations exist and the oncological RECIST criteria may not be the best option for assessing vascular malformations. In this regard, new functional imaging modalities and advanced analysis tools may prove more versatile for treatment response evaluation in the future (30, 31). These aspects emphasize the general complexity in studying this rare disease, as there are currently no established criteria for the analysis of both clinical and objective response. Fourth, in regard of the high recurrence rate of VMs commonly manifesting after a longer-term, the mean follow-up time of about 9 months after the last sclerotherapy was relatively short. Therefore, it was not yet reasonable to evaluate the recurrence rate as an essential outcome parameter in vascular anomalies. Consequently, conclusions with respect to long-term efficacy of the proposed approach were not feasible.

## Conclusion

Image-guided sclerotherapy is effective for treating venous malformations of the face. Clinical response is not always associated with lesion size reduction on imaging. Despite the challenging and complex anatomy of this location, the procedures carry low complication rates for both adults and children.

## Data availability statement

The raw data supporting the conclusions of this article will be made available by the authors, without undue reservation.

## Ethics statement

The studies involving human participants were reviewed and approved by the Local Ethics Committee (University Hospital, LMU Munich, protocol No. 21-0943, 10/06/2021). Written informed consent from the participants’ legal guardian/next of kin was not required to participate in this study in accordance with the national legislation and the institutional requirements. Written informed consent was obtained from the minor(s)’ legal guardian/next of kin for the publication of any potentially identifiable images or data included in this article.

## Author contributions

VS, MM, and MW contributed to the conception and design of the study. VS contributed to the organization of the database, performed the statistical analysis, and wrote the first draft of the manuscript. MM, CG, PS, LS, VS-Z, SD, OÖ, NM, MÜ, MDS, BB, EM, AN, and OU contributed to the acquisition of the data. RB, MK, MS, JR, BG, and WW contributed to the interpretation of the data. All authors revised the work critically for important intellectual content and provided approval for publication of the content, contributed to the article, and approved the submitted version.
